# In Situ Chemically-Selective Monitoring of Multiphase Displacement Processes in a Carbonate Rock Using 3D Magnetic Resonance Imaging

**DOI:** 10.1007/s11242-017-0945-6

**Published:** 2017-11-13

**Authors:** N. P. Ramskill, A. J. Sederman, M. D. Mantle, M. Appel, H. de Jong, L. F. Gladden

**Affiliations:** 1 Department of Chemical Engineering and Biotechnology, University of Cambridge, West Cambridge Site, Philippa Fawcett Drive, Cambridge, CB3 0AS UK; 2 Shell Technology Centre, 3333 Highway 6 S, Houston, TX USA

**Keywords:** Compressed sensing, MRI, Chemically-selective imaging

## Abstract

Accurate monitoring of multiphase displacement processes is essential for the development, validation and benchmarking of numerical models used for reservoir simulation and for asset characterization. Here we demonstrate the first application of a chemically-selective 3D magnetic resonance imaging (MRI) technique which provides high-temporal resolution, quantitative, spatially resolved information of oil and water saturations during a dynamic imbibition core flood experiment in an Estaillades carbonate rock. Firstly, the relative saturations of dodecane ($S_{\mathrm{o}})$ and water ($S_{\mathrm{w}})$, as determined from the MRI measurements, have been benchmarked against those obtained from nuclear magnetic resonance (NMR) spectroscopy and volumetric analysis of the core flood effluent. Excellent agreement between both the NMR and MRI determinations of $S_{\mathrm{o}}$ and $S_{\mathrm{w}}$ was obtained. These values were in agreement to 4 and 9% of the values determined by volumetric analysis, with absolute errors in the measurement of saturation determined by NMR and MRI being 0.04 or less over the range of relative saturations investigated. The chemically-selective 3D MRI method was subsequently applied to monitor the displacement of dodecane in the core plug sample by water under continuous flow conditions at an interstitial velocity of $1.27\times 10^{-6}\,\hbox {m}\,\hbox {s}^{-1}$ ($0.4\,\hbox {ft}\,\hbox {day}^{-1})$. During the core flood, independent images of water and oil distributions within the rock core plug at a spatial resolution of $0.31\,\hbox {mm}\times 0.39\,\hbox {mm} \times 0.39\,\hbox {mm}$ were acquired on a timescale of 16 min per image. Using this technique the spatial and temporal dynamics of the displacement process have been monitored. This MRI technique will provide insights to structure–transport relationships associated with multiphase displacement processes in complex porous materials, such as those encountered in petrophysics research.

## Introduction

Oil provides 32.9% of global energy consumption (British Petroleum Company 2016), and there, therefore, exists strong motivation to derisk the exploration and improve the efficiency of production of oil and gas, through a better understanding and optimization of the hydrocarbon recovery process. One approach to achieving this aim has been in the development of so-called digital rock (DR) technology, which combines high-resolution digital imaging of rock samples with the numerical simulation of the hydrodynamics in the pore space (Blunt et al. 2013; Koroteev et al. 2014). To date the DR approach has focused on the understanding of fluid–fluid displacement processes, but the longer-term ambition for the method is to inform production strategies in the field. However, before these numerical simulators can be deployed into practical applications, their predictive capabilities must be validated against experimental studies on both the pore and core length scales. It follows that experimental methods capable of providing dynamic, quantitative and spatially resolved information are therefore required.

Currently, magnetic resonance imaging (MRI) and X-ray computed tomography (CT) are the most widely used techniques for imaging in situ core flood fluid distributions, both of which can non-destructively image multiphase fluid systems in porous media (Mitchell et al. 2013a). In previous works, X-ray-based methods have been used to great effect to investigate multiphase displacement processes both on the pore scale with X-ray microtomography ($\upmu \hbox {CT}$) (Berg et al. 2014; Andrew et al. 2013; Rücker et al. 2015; Schmatz et al. 2015; Berg et al. 2016) and on the core scale with X-ray medical CT (Pini et al. 2012; Krevor et al. 2012). With X-ray $\upmu$CT, the typical core plug sample size that can be studied is approximately 6.4 mm diameter, which poses an inherent limitation on the length scales over which the displacements can be observed. Although X-ray $\upmu \hbox {CT}$ is capable of producing images at a higher spatial resolution ($10^{0} \,\upmu \hbox {m}\,\hbox {voxel}^{-1})$ than medical CT and MRI ($10^{2} \, \upmu \hbox {m}\,\hbox {voxel}^{-1})$, the latter two methods can be used to study much larger core plugs typically between 25 and 50 mm diameter and are therefore well suited to investigating structure–transport relationships on the core scale. Further, direct measurement of molecular diffusion and flow within rock cores using magnetic resonance is well established (Mitchell et al. 2008; Colbourne et al. 2016). Considering the relative attributes and limitations of the two imaging modalities, there are great opportunities to exploit the synergy of the two techniques to provide complementary information, thereby enabling the holistic characterization of the multiphase displacement processes that are of interest, from the pore to the core scale. In the present study, the focus has been on the development and application of an MRI technique that can be applied to provide quantitative, spatially resolved information on the distributions of the hydrocarbon and aqueous phases during dynamic core flood experiments.

A particular advantage of using MRI is that there is a range of non-invasive strategies for providing contrast between the different fluid phases in the sample without the need to add dopants, as is often necessary with X-ray CT. These non-invasive fluid contrast mechanisms that may be exploited are: detection of specific NMR-active nuclei ($$^{1}\hbox {H}$$, $$^{13}\hbox {C}$$, $$^{23}\hbox {Na}$$, etc.), spectroscopic chemical shift sensitivity, longitudinal and transverse relaxation time ($$T_{1}$$ and $$T_{2})$$ weighting and diffusivity contrast. The choice of the contrast mechanism to be utilized in the imaging experiment will depend on the nature of the system under investigation and the information required. Examples of the applications of these approaches to studying rock cores are now given.

It has been demonstrated that $^{23}\hbox {Na}$ MRI can be used to provide direct images of the location of brine solution using $^{23}\hbox {Na}$ observation (Washburn and Madelin 2010; Mitchell and Fordham 2014). Of relevance to the present study, Washburn and Madelin (2010) have demonstrated a $^{23}\hbox {Na}$ MRI method to monitor the movement of brine into an oil-saturated Bentheimer sandstone during a spontaneous imbibition process; however, no direct information on the hydrocarbon phase can be obtained. For systems in which the contributions from the hydrocarbon and aqueous phases can be resolved in the same NMR spectrum, it has been shown that the chemical shift sensitivity can be used to isolate the signal from the two phases independently of one another. A requirement of this approach is that the peaks in the NMR spectrum must be well resolved with little overlap to enable the accurate discrimination of the different chemical species present in the sample. Dechter et al. (1989, 1991) have shown that by using a pre-saturation method, in which the NMR signal from one of the phases is suppressed, fluid-specific images of refined oil and aqueous brine in carbonate (Dolomite) and sandstone (Bentheimer) cores can be obtained. This technique was found to be particularly effective in core plug samples with a low paramagnetic content, such as certain carbonates, and less effective in clay-rich samples where chemical shift separation is more challenging due to spectral line broadening induced by magnetic susceptibility gradients associated with the rock. In addition to the type of rock under investigation, the nature of the fluids within the pores must also be considered to determine whether the spectral peaks associated with the hydrocarbon and aqueous phases can be separated in the NMR spectrum. More specifically, the relatively complex chemical composition of crude oil is expected to result in broader peaks in the NMR spectrum than compared with, for example, alkanes or refined oils, which are often used as a proxy for the hydrocarbon phase in laboratory core analysis.

An alternative approach to discriminating between hydrocarbon and aqueous phases is to exploit differences in their respective NMR relaxation times or diffusion coefficients. One such approach is the inversion-nulling method in which species can be differentiated from one another based on their $T_{1}$ as has been demonstrated by Hall and Rajanayagam (1987) for the imaging of oil and water in sandstones. Chemically specific images of oil and water have also been obtained from $T_{1}$ or $T_{2}$ maps by integrating the signal intensity across different ranges of the relaxation time spectrum and assigning to a particular fluid phase (Xiao and Balcom 2012; Mitchell et al. 2012, 2013b). Liu et al. (2015) have also successfully demonstrated the identification of oil and brine in a rock during a core flood using spatially resolved $D-T_{2}$ imaging in one spatial dimension.

To study multiphase displacement processes in rock cores, 3D imaging is desirable in order to visualize the total fluid saturation within the sample. However, the acquisition times for relatively high spatial resolution 3D images using traditional MRI techniques, such as spin-warp (Edelstein et al. 1980) or pure phase-encoded techniques (Parasoglou et al. 2009; Petrov and Balcom 2011; Xiao and Balcom 2012, 2013), are too long to capture the dynamic nature of the displacement mechanisms that are of interest at representative reservoir interstitial velocities. Xiao and Balcom (2015) and Li et al. (2016) have recently reported the implementation of $\pi$-EPI to decrease data acquisition times in application to 3D fluid saturations during dynamic core flood experiments. Ramskill et al. (2016) used an alternative approach and employed the rapid acquisition with relaxation enhancement (RARE) pulse sequence (Hennig et al. 1986) combined with compressed sensing (CS) (Lustig et al. 2007, 2008; Benning et al. 2014) to record 3D images of the fluid distribution in rock core plugs in $\sim 16$ min and with an isotropic spatial resolution of $\sim 390\,\upmu \hbox {m}$. In the present work, this method is extended to acquire chemically specific images of the hydrocarbon and aqueous phases independently of one another. To retain the quantitative nature of the magnetic resonance experiment, the implementation of the method has to be considered carefully, as is now discussed.

Whilst it is well known that, in principle, the signal intensity in a magnetic resonance experiment is proportional to the number of NMR-active species present, consideration must be given as to how the data are acquired and analysed depending on the methods used if the quantitative nature of the measurement is to be retained; examples of such work in the field of fluids in porous media include Mantle (2011), Mitchell et al. (2012) and Li et al. (2016). The challenge arises because signal attenuation occurs in the dead time between excitation and detection of the nuclear spins due to spin relaxation. For NMR spectroscopy measurements, using the pulse-acquire method, the dead time is typically very short (on the order of microseconds) and attenuation due to relaxation will be negligible for the rock–fluid systems of interest in this work. It follows that the acquired NMR signal intensity can therefore be considered a true representation of the spin density, i.e. the amount of fluid present in the volume of interest. However, in MRI experiments, due to the time required for spatial encoding using magnetic field gradients, longer dead times on the order of milliseconds are common and relaxation effects cannot be ignored. For multicomponent systems in porous media, this is further complicated by the fact that different chemical species are often characterized by different relaxation times depending on their interaction with the solid surface, which is a manifestation of the wettability and pore size in which the spins reside. Consequently, the signal intensity associated with the different chemical species will contain varying degrees of relaxation weighting, which must be considered when quantifying the relative amounts of each phase within the system. In the context of the present work, a routine implementation of the RARE imaging sequence is not considered to be quantitative due to the effect of relaxation that occurs during the echo train (Hennig et al. 1986; Mantle 2011). Following the excitation pulse, the signal will relax according to a $T_{2}$-like decay, which is actually governed by a coherent superposition of $T_{2}$-weighted spin echoes and $T_{1}$-weighted stimulated echoes. Ultimately this produces images in which the intensity contains a complex combination of both $T_{1}$ and $T_{2}$ weighting thus making quantitation from these measurements difficult (Hennig et al. 1986). However, it has been shown that quantitative maps of the moisture content in porous materials can be obtained using the RARE pulse sequence by acquiring multiple $T_{2}$-weighted images and subsequently fitting the pixel intensity for each image to an exponential decay function to determine the local intensity without $T_{2}$ weighting (Chen et al. 2010). Unfortunately, the need to acquire additional $T_{2}$-weighted images increases the data acquisition time making the approach unsuitable for studying a dynamic process such as the core flood of interest here. Instead, we propose and demonstrate a strategy to minimize the effect of $T_{2}$ relaxation in a single image acquisition whilst retaining an image acquisition time of $\sim 16\,\hbox {min}$.

In the present work, we demonstrate the first application of an MRI technique capable of providing high-temporal resolution, independent images of both the hydrocarbon and aqueous phases in the same rock system during a dynamic core flood experiment. By combining the CS-RARE pulse sequence (Ramskill et al. 2016) with chemically-selective preconditioning (Dechter et al. 1989, 1991), independent 3D images of the oil and water phases have been acquired in $\sim 16$ min, thus enabling the dynamics of the displacement process to be observed. Given the injection rate of $0.025\,\hbox {ml}\,\hbox {min}^{-1}$, this corresponds to an injection of less than 4% of the total pore volume of the rock over the course of the acquisition of both the hydrocarbon and water distribution images. Using this technique, we show that the relative saturations of the hydrocarbon and aqueous phases can also be monitored quantitatively throughout the core flood experiment.

## Materials and Methods

Experiments were performed as follows: (i) optimization and validation of the chemically-selective 3D MRI technique is demonstrated on an ‘inject-stop-acquire’ core flood experiment and (ii) application of the chemically-selective 3D MRI technique to monitor the displacement of a hydrocarbon by an aqueous injectant under continuous flow conditions. For both sets of experiments the same Estaillades limestone core plug, 38 mm in diameter and 68 mm in length, was used as a representative sample of a hydrocarbon-bearing reservoir rock. After drying the rock at $95\,^{\circ }\hbox {C}$ for 24 h and upon vacuum saturation with dodecane, the pore volume (P.V.) was determined by gravimetric analysis to be $22.35\pm 0.03\,\hbox {ml}$, thus corresponding to a porosity of  $\phi \,=\,29\pm 2\%$. For core flood experiments, the sample was held in an Aflas sleeve within a PEEK rock core holder (ErgoTech, Conwy, UK), which was placed within the imaging region of the magnet. A constant confining pressure was applied to the outside of the Aflas sleeve by per-fluorinated oil (Fluorinert FC-43) using a Gilson 307 (Gilson Inc., USA) HPLC pump maintained at $1.7\pm 0.2$ MPa by a back pressure regulator (IDEX Health and Science, USA). For the injection of deionized water, a Quizix QX-1500 pump (Chandler Engineering, USA) has been used. All MR experiments were carried out on a 2 T (85 MHz for $^{1}$H) horizontal-bore magnet controlled by a Bruker AV spectrometer. A 60 mm radio frequency (r.f.) coil tuned to a frequency of 85.2 MHz was used for excitation and signal detection. Spatial resolution was achieved using magnetic field gradients with a maximum strength of $10.7~\hbox {G}\,\hbox {cm}^{-1}$. The specific details pertaining to each of the two sets of experiments will now be described.

### Implementation of Quantitative Chemically-Selective 3D MRI

The aim of the inject-stop-acquire core flood experiment was to optimize and subsequently validate the chemically-selective 3D MRI technique to ensure that quantitative measurements of the relative saturations of the dodecane and water within the core plug sample are obtained. A suite of NMR spectroscopy, MRI and NMR relaxation time measurements were conducted, following the injection of 5.8, 11.6 and 17.4 ml of deionized water into the dodecane-saturated sample at a flow rate of $0.05\,\hbox {ml}\,\hbox {min}^{-1}$. Between each injection stage, the flow was stopped and the volumes of dodecane and water produced in the effluent were recorded. The total measurement time for the suite of magnetic resonance measurements at each injection stage was approximately 10 h. The implementation details of each of the magnetic resonance techniques used are now described.

#### NMR Spectroscopy

**Fig. 1:**
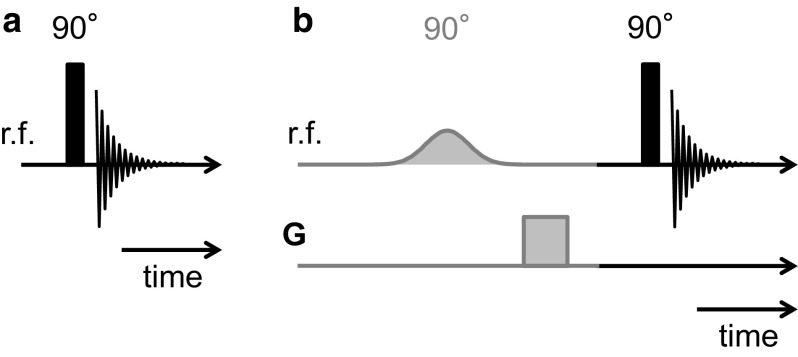
Pulse sequences used for the **a** non-chemically-selective and **b** chemically-selective NMR spectroscopy measurements. The solid vertical bars are the non-selective r.f. pulses. The grey shaded section in **b** indicates the chemically-selective preconditioning stage, where the selective Gaussian-shaped pulses and homospoil gradients (**G**) are used to selectively excite and then suppress specific regions of the NMR spectrum

As discussed previously, NMR spectra are regarded as being a quantitative measurement of the amount of the chemical species present in the sample and have therefore been used to benchmark the saturation values obtained from the MRI measurements. The sample used to set the implementation parameters was the dodecane-saturated core plug into which 5.8 ml of water had been injected. Firstly, standard spectra (i.e. with no chemical selection) were acquired using the pulse-acquire technique for which the pulse sequence diagram is shown in Fig. 1a. The duration of the $90^{\circ }$ hard r.f. excitation pulse was $20\,\upmu \hbox {s}$, and a free induction decay (FID) time domain signal was acquired at a sampling rate of 20 kHz which is then Fourier transformed to obtain the NMR spectrum. Chemically-selective spectra were then acquired using the pre-conditioned pulse-acquire technique (Fig. 1b). Gaussian-shaped $90^{\circ }$ excitation pulses with a duration $8192\, \upmu \hbox {s}$ and an associated frequency bandwidth of approximately 190 Hz (full width at half maximum) were used. By applying the selective pulse at a particular offset frequency, specific regions of the NMR spectrum, corresponding to either the water or dodecane in the rock sample, are selectively excited. Homospoil gradients (grey shaded boxes) in three orthogonal directions are subsequently applied for 2 ms at a strength of $\mathbf{G} = 5.3\,\hbox {G}\,\hbox {cm}^{-1}$ to eliminate any signal associated with the spins excited by the selective pulse. Thus, the residual magnetization available for detection is due to the spins unaffected by the selective pulse. The optimized offset frequencies for the chemically-selective pulses were found to be at $\delta = 1.3\,\hbox {ppm}$ and $\delta = 4.8\,\hbox {ppm}$ for suppression of the dodecane and water, respectively. The successful implementation of the chemically-selective pulse sequence is demonstrated in Sect. 3.1.1. The same parameters are used in all chemically-selective spectroscopy and MRI measurements reported.

#### Magnetic Resonance Imaging

**Fig. 2:**
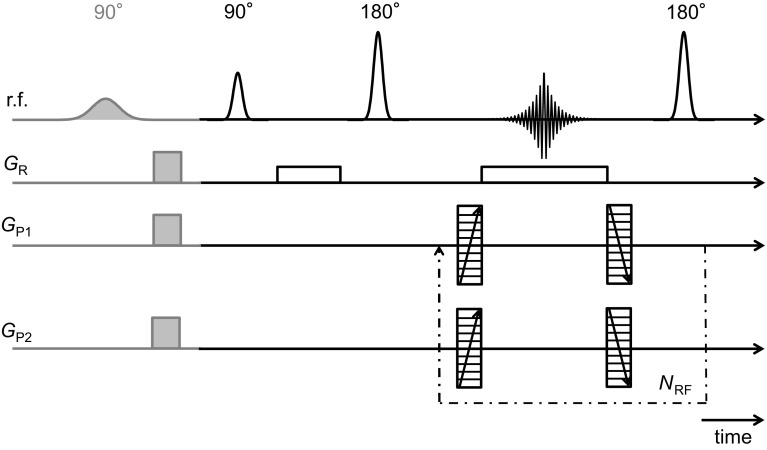
Schematic of the rapid acquisition with relaxation enhancement (RARE) pulse sequence. **k**-space is frequency encoded in the read direction ($k_{\mathrm{R}})$ and phase encoded in $k_{\mathrm{P1}}$ and $k_{\mathrm{P2}}$ directions upon the application of the read gradient ($G_{\mathrm{R}})$ and phase gradients ($G_{\mathrm{P1}}$ and $G_{\mathrm{P2}})$, respectively. The number of $180^{\circ }$ degree pulses applied for each acquisition is determined by the RARE factor ($N_{\mathrm{RF}})$. The grey shaded section indicates the chemically-selective preconditioning stage of the pulse sequence

Figure 2 shows the RARE pulse sequence, which has been used as the basis for the imaging experiments. In the pulse sequence diagram, the grey shaded section is the preconditioning stage used for the chemically-selective image acquisitions. At each stage in the inject-stop-acquire core flood experiment, fully sampled and under-sampled 3D images both without and with chemically-selective excitations were acquired. Herein, RARE refers to the conventional implementation of the RARE pulse sequence to acquire fully sampled images and CS-RARE refers to the method by which **k**-space is under-sampled and compressed sensing (CS) is used for the image reconstruction (Ramskill et al. 2016). Gaussian-shaped $90^{\circ }$ excitation and $180^{\circ }$ refocusing r.f. pulses of duration of $256\,\upmu \hbox {s}$ and power levels of 20 and 14 dB, respectively, were used. For the chemically-selective preconditioning, the same experimental parameters detailed in Sect. 2.1.1. were used. Typical echo times for the RARE acquisitions were approximately $T_{\mathrm{E}}\,=\,5\,\hbox {ms}$. All images were acquired with a field-of-view (FOV) of $80\,\hbox {mm}\,\times \,50\,\hbox {mm}\,\times \,50\,\hbox {mm}$ in the *z*, *x* and *y* directions, respectively, and for a data matrix size of $256\,\times \,128\,\times \,128$ pixels, which gave a nominal image resolution of $0.31\,\hbox {mm}~\times ~0.39\,\hbox {mm}\,\times \,0.39\,\hbox {mm}$. For all experiments a RARE factor of $N_{\mathrm{RF}} = 32$ was used which means that a train of 32 echoes are acquired for each excitation of the system. With four scans for signal averaging and a recycle delay of 6 s, the acquisition times for the RARE and CS-RARE images were approximately 3.5 h and 50 min, respectively. For the CS-RARE acquisitions, two **k**-space under sampling approaches, both at 25% sampling, have been tested (Sect. 3.1.2). The interested reader is referred to Ramskill et al. (2016) for the details associated with the design of the sampling pattern used in the under-sampled acquisitions. The compressed sensing image reconstructions were carried out using an in-house MATLAB toolbox, Object Oriented Mathematics for Inverse Problems (OOMFIP) for which the implementation is presented in Benning et al. (2014) and image post-processing was performed in Avizo Fire 8.1 (FEI Visualisation Sciences Group, USA).

For completeness, RARE and CS-RARE images were also acquired with a shorter recycle delay of 2 s, resulting in acquisition times of 70 and 16 min, respectively, in order to confirm that the quantitative character of the data is retained with the shorter recycle delay which is used for the dynamic core flood experiments (Sect. 2.2); in particular, these experiments confirm that the dynamic core flood data are not affected by significant $T_{1}$-relaxation.

#### NMR Relaxation Time Analysis

**Fig. 3:**
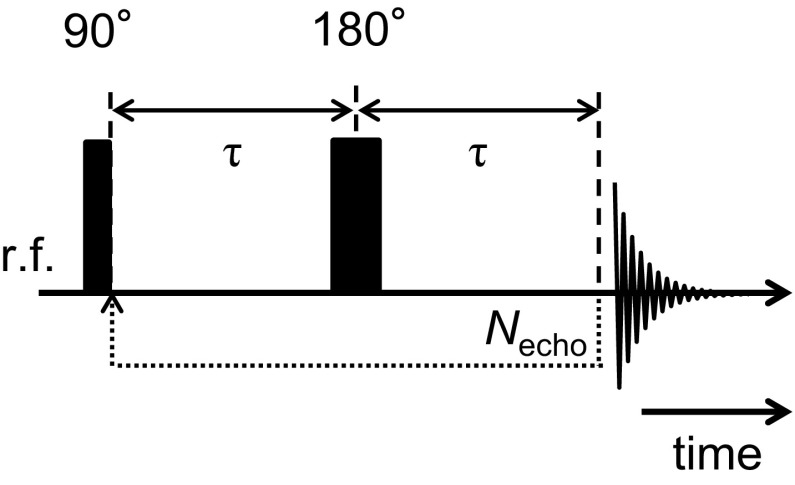
Pulse sequence used for the CPMG $T_{2}$ measurements. A series of NMR spectra are acquired with varying degrees of $T_{2}$ weighting by varying the number of $180^{\circ }$ r.f. pulses, $N_{\mathrm{echo}}$, prior to the acquisition of the FID

The $T_{2}$ of the dodecane and water in the rock were measured to provide the constraints for the design of the optimal **k**-space sampling protocol, used for the CS-RARE acquisitions. The $T_{2}$ relaxation times were measured using the Carr–Purcell–Meiboom–Gill (CPMG) (Carr and Purcell 1954; Meiboom and Gill 1958) method, shown by the pulse sequence diagram in Fig. 3. For these measurements, the duration of the $90^{\circ }$ and $180^{\circ }$ hard pulses was 20 and $40\,\upmu \hbox {s}$, respectively, and FID signals were acquired at a sampling rate of 20 kHz. FID signals were collected for 32 different delay times ranging from 10 ms to 5 s, determined by the number of $180^{\circ }$ pulses ($N_{\mathrm{echo}})$ applied in the echo train where the echo time, $T_{\mathrm{E}}$ ($2\tau )$, was 1.5 ms. With 8 scans for signal averaging, the acquisition time was approximately 45 min. The time domain FID signals were subsequently Fourier transformed to yield NMR spectra with different amounts of $T_{2}$ weighting, and the relaxation time analysis was performed for the dodecane and water signals separately. The $T_{2}$ attenuation data (*m*) are described by the first-order Fredholm integral equation given by Eq. 1:

1 $$\begin{aligned} \frac{m\left( {N_{\mathrm{echo}} T_\mathrm{E} } \right) }{m\left( 0 \right) }=\int _{0}^{\infty } k\left( {N_{\mathrm{echo}} T_\mathrm{E} ,T_2 } \right) f\left( {\log _{10} T_2 } \right) d\log _{10} T_2 +\epsilon , \end{aligned}$$

where $\epsilon$ is the experimental noise and *k* is the kernel function describing the transverse relaxation:

2 $$\begin{aligned} k=\hbox {exp}\left\{ {-\frac{N_{\mathrm{echo}} T_\mathrm{E} }{T_2 }} \right\} . \end{aligned}$$

It is commonly accepted that liquids within a porous medium, such as a rock core, will exhibit a distribution of relaxation times, which is determined by the surface area to volume ratio of the pores and the surface relaxivity of the material (Mitchell et al. 2013a). In the present study, the distribution of ${T}_{2}$ values in the sample was obtained following the inversion method of Venkataramanan (2002) using Tikhonov regularization (Butler et al. 1981) with the optimization of the regularization parameter performed using the generalized cross-validation (GCV) method (Wahba 1977).

### Continuous Displacement Core Flood Experiment

For the continuous displacement core flood experiment, deionized water was injected into the dodecane-saturated core plug at a flow rate of $0.025\,\hbox {ml}\,\hbox {min}^{-1}$ corresponding to an interstitial velocity of $1.27\,\times 10^{-6}\,\hbox {m}\,\hbox {s}^{-1}$ ($0.4\,\hbox {ft}\,\hbox {day}^{-1})$, based on the cross-sectional area of the plug and the bulk porosity of the rock. During this process, three NMR spectra (one standard spectrum and two spectra, chemically-selective to each of water and dodecane) with the same experimental parameters as detailed in Sect. 2.1.1, and chemically-selective 3D CS-RARE measurements of the water and dodecane were acquired in series. Again, all images were acquired with a FOV of $80\,\hbox {mm}\,\times \,50\,\hbox {mm}\,\times \,50\,\hbox {mm}$ in the *z*, *x* and *y* directions, respectively, and a data matrix size of $256\,\times \,128\,\times \,128$ pixels, which gave a nominal image resolution of $0.31\,\hbox {mm}\,\times ~0.39\,\hbox {mm}\,\times \,0.39\hbox { mm}$.

With a RARE factor of $N_{\mathrm{RF}} = 32$, four scans for signal averaging and a recycle delay of 2 s, the acquisition time for a single chemically-selective image was 16 min; thus, the time taken to acquire both the chemically-selective dodecane and water images is 32 min. The shorter recycle delay of 2 s was used for the chemically-selective MRI measurements for the continuous flow experiments in order to more accurately resolve the dynamics of the oil and water distributions during the core flood. The acquisition of the spectra and chemically-selective MRI measurements were acquired in series until 1.2 P.V. of water had been injected into the rock and the oil production had plateaued.

## Results and Discussion

### Inject-Stop-Acquire Core Flood: Validation of the Quantitative Nature of the Technique

#### NMR Spectroscopy

**Fig. 4:**
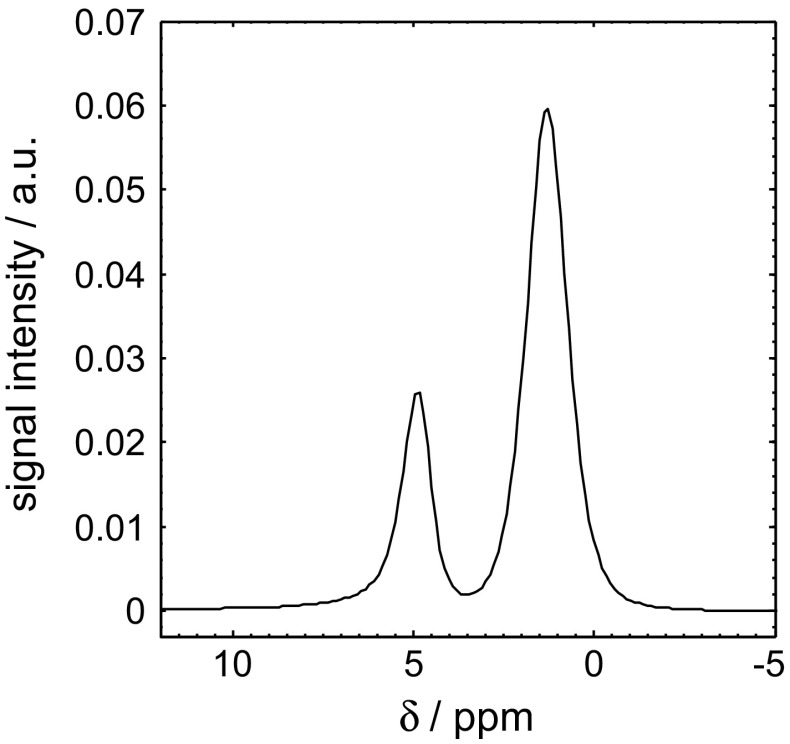
NMR spectrum of water ($\delta = 4.8\,\hbox {ppm}$) and dodecane ($\hbox {CH}_{2} \quad \delta = 1.3\,\hbox {ppm}$) following the injection of 5.8 ml of water into the initially dodecane-saturated Estaillades core plug sample. Chemical shift references are given relative to the $^{1}\hbox {H}$ resonance of tetramethylsilane

**Fig. 5:**
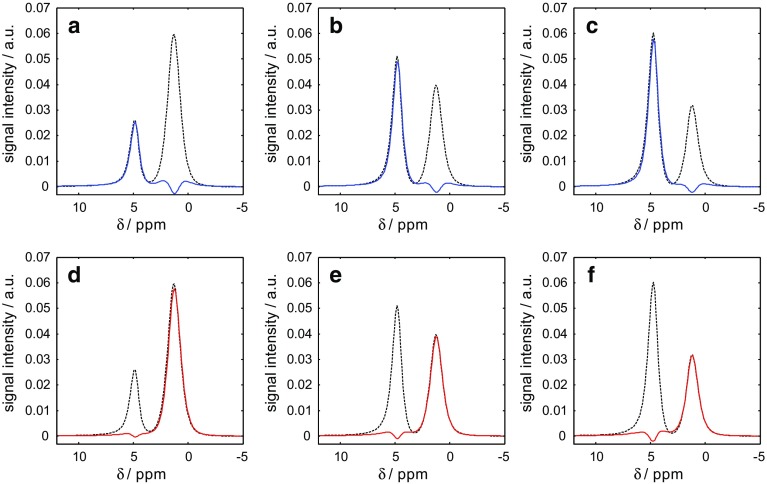
Series of NMR spectra acquired during the ‘inject-stop-acquire’ core flood experiments at 5.8 ml (**a**, **d**), 11.6 ml (**b**, **e**) and 17.4 ml (**c**, **f**) of water injected. The spectra acquired using the non-chemically-selective pulse-acquire method are shown as the dashed lines in background, and those acquired using the chemically-selective pulse-acquire method are shown as red (dodecane) and blue (water) solid lines in the foreground

In order to demonstrate the quantitative nature of the MRI technique, the relative oil ($S_{\mathrm{o}})$ and water ($S_{\mathrm{w}})$ saturations in the rock core plug during the core flood experiment were benchmarked against those obtained using NMR spectroscopy. Figure 4 shows the standard NMR spectrum acquired following the injection of 5.8 ml of water into the initially dodecane-saturated core plug. From this, it can be seen that the water ($\delta = 4.8\, \hbox {ppm}$) and dodecane ($\delta = 1.3\,\hbox {ppm}$) peaks in the spectrum are well resolved with very little overlap; these peak positions identify the optimized offset frequencies of $\delta = 1.3\,\hbox {ppm}$ and $\delta = 4.8\,\hbox {ppm}$ for suppression of the dodecane and water, respectively. Using these offset frequencies along with the other pulse sequence implementation parameters for chemically-selective acquisition reported in Sect. 2.1.1, the data shown in Fig. 5 are obtained for the 3 volumes of water injected into the initially dodecane-saturated core plug. Figure 5 shows the NMR spectra acquired with the standard pulse-acquire method overlaid with those acquired using the pulse acquire with chemically-selective preconditioning method following the injection of 5.8 ml (Fig. 5a, d), 11.6 ml (Fig. 5b, e) and 17.4 ml (Fig. 5c, f) of water.

**Fig. 6:**
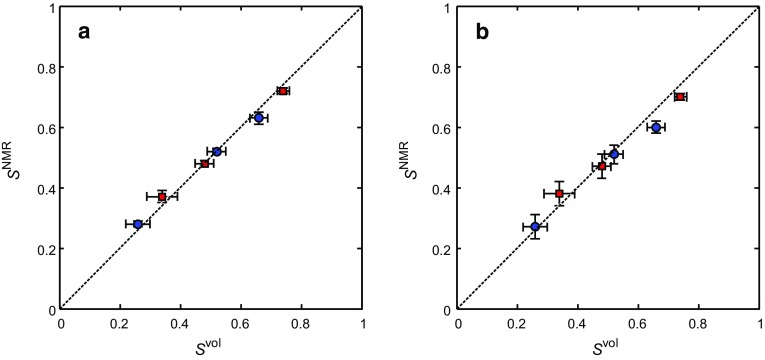
Comparison of the NMR and volumetric determinations of the relative saturations of the dodecane $S_{\mathrm{o}}$ (red squares) and water $S_{\mathrm{w}}$ (blue circles) from the NMR spectra acquired using the **a** non-chemically-selective and **b** chemically-selective pulse-acquire methods

From the NMR spectra acquired using the chemically-selective preconditioning method, it is seen that the signal associated with the dodecane (Fig. 5a–c) and water (Fig. 5d–f) is effectively suppressed by the preconditioning stage, whilst there is negligible influence on the signal associated with the spins intended to be unaffected by the selective pulse. It is clearly seen that the signal associated with the water increases, whilst that from the dodecane decreases as the core flood proceeds. The relative saturations $S_{\mathrm{w}}$ and $S_{\mathrm{o}}$ are determined from the NMR spectroscopy data shown in Fig. 5 using Eqs. 3 and 4, respectively:

3 $$\begin{aligned} S_{\mathrm{w}}= & {} \frac{\mathrm{HI}\cdot I_{{\text {MR}{-}\mathrm{W}}}}{\mathrm{HI}\cdot I_{{{\text {MR}{-}\mathrm{W}}}} +I_{{{\text {MR}{-}\mathrm{O}}} }} \end{aligned}$$

4 $$\begin{aligned} S_{\mathrm{o}}= & {} \frac{I_{{{\text {O}{-}\mathrm{MR}}} }}{\mathrm{HI}\cdot I_{{\text {MR}{-}\mathrm{W}}} +I_{{{\text {MR}{-}\mathrm{O}}} }} \end{aligned}$$

where $I_{\mathrm{MR-W}}$ and $I_{\mathrm{MR-O}}$ are the integrated signal intensities of the water and dodecane as determined from the spectra and HI is the hydrogen index of the dodecane, which was determined experimentally to be $\mathrm{HI }= 1.03\pm 0.01$. The signal intensity associated with the water and dodecane was obtained by integrating the area upfield and downfield of a cut-off located at 3.5 ppm, respectively. Figure 6 shows the relative saturations of the dodecane and water as determined by the non-chemically-selective and chemically-selective NMR spectroscopy methods compared with those determined by volumetric analysis of the core flood effluent.

**Fig. 7:**
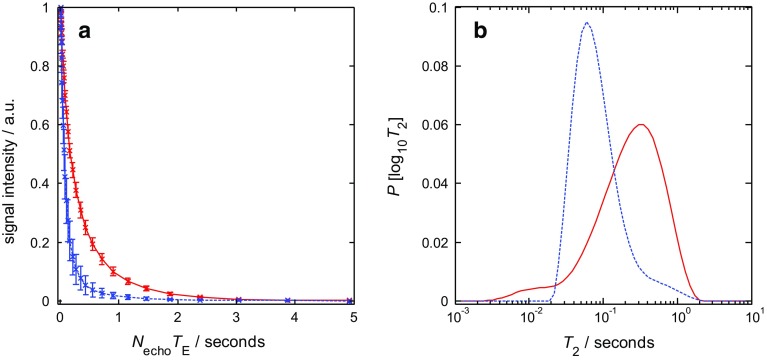
**a** Average normalized attenuation data from the CPMG $T_{2}$ measurements for the water (blue dashed line) and dodecane (red solid line). The error bars are the standard deviation from the measurements conducted at the three stages of the inject-stop-acquire core flood experiment. **b** The distributions of $T_{2}$ for the water (blue dashed line) and dodecane (red solid line) in the rock as obtained from the numerical inversion of the attenuation data shown in **a**

From Fig. 6, it is seen that the saturation values as determined by the non-selective (Fig. 6a) and chemically-selective (Fig. 6b) NMR methods are in agreement with those obtained from the volumetric methods to within 4 and 6%, respectively, over the range of saturations investigated. The absolute error in the saturation measurements, $\varDelta S$, of the non-selective and chemically-selective measurements are 0.02 and 0.03, respectively. The values quoted are an average over all saturations. This therefore demonstrates that the NMR spectroscopy measurements are suitable for benchmarking the MRI determinations of the saturation and that the chemically-selective preconditioning is retaining the quantitative nature of the magnetic resonance measurement.

#### Magnetic Resonance Imaging

The optimization of the CS-RARE **k**-space sampling protocol to minimize the amount of $T_{2}$ relaxation weighting in the image and therefore to enable acquisition of images in which the signal intensity remains a quantitative measure of the fluid content in the rock is now described. To provide relevant information to inform the selection of a suitable sampling pattern, the $T_{2}$ relaxation times of the dodecane and water in the Estaillades were measured as shown in Fig. 7. Figure 7a shows the averaged attenuation data from the $T_{2}$ experiments conducted at the three stages of the inject-stop-acquire core flood experiment. Figure 7b shows the distributions $T_{2}$ for the water and dodecane in the rock as obtained from the numerical inversion of the attenuation data shown in Fig. 7a. The numerical inversion was performed on the average attenuation data (Fig. 7a) in order to determine the relaxation times of the dodecane and water across the range of saturation states that are relevant to the dynamic displacement experiment presented in Sect. 3.2. From Fig. 7b it is seen that the water is generally characterized by shorter values of $T_{2}$ than the dodecane, with the logarithmic mean $T_{2}$ ($T_{2\mathrm{LM}})$ being approximately $T_{2\mathrm{LM}}= 80\,\hbox {ms}$, for the former and approximately $T_{2\mathrm{LM}} = 200\,\hbox {ms}$, for the latter.

**Fig. 8:**
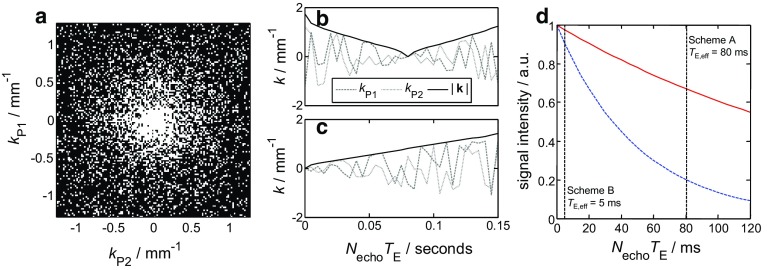
**a** Typical variable density **k**-space sampling pattern used for the CS-RARE acquisitions for 25% sampling where the white pixels indicate the regions of **k**-space that are sampled. **b** Scheme A and **c** Scheme B show the trajectories for the two phase-encoding gradients ($k_{\mathrm{P1}}$ and $k_{\mathrm{P2}})$ for a single echo train ($N_{\mathrm{echo}} = 32$) for the two **k**-space sampling methods that have been tested. **d** Simulated signal attenuation of the water (blue dashed line) and dodecane (red solid line) according to the model for $T_{2}$ relaxation (Eq. 2) during a single echo train of a RARE experiment. The point at which $\mathbf{k} = 0\,\hbox {mm}^{-1}$ is sampled for each of the **k**-space sampling methods (Schemes A and B) is indicated by the vertical dashed lines. Values of the log mean $T_{2}$ have been used for the simulated decay where $T_{\textsc {2LM}}=80\,\hbox {ms}$ and $T_{\textsc {2LM}} = 200\,\hbox {ms}$ for the water and dodecane, respectively

Figure 8a shows a typical sampling pattern that has been used in the CS-RARE acquisitions in the present study for 25% sampling of **k**-space where the white pixels are the lines of **k**-space that are fully sampled (into the page) (Ramskill et al. 2016). Two **k**-space sampling approaches were tested for the CS-RARE acquisitions for which the **k** values sampled during a single echo train in the RARE acquisition ($N_{\mathrm{echo}}= 32$) are shown in Fig. 8b (Scheme A) and 8c (Scheme B), where the echo time, $T_{\mathrm{E}} = 5\,\hbox {ms}$. It is the signal amplitude at the centre of **k**-space $(\mathbf{k} = 0\,\hbox {mm}^{-1})$ that contains the information for the overall signal intensity in the image. Therefore, it is the time elapsed between the $90^{\circ }$ excitation pulse and the acquisition of the signal at the centre of **k**-space that determines the extent of signal attenuation due to relaxation, and hence the loss of quantitation in the acquired signal; this elapsed time is referred to as the effective echo time, $T_{\mathrm{E,eff}}~=~N_{\mathrm{echo}}T_{\mathrm{E}}$ where $N_{\mathrm{echo}}$ is the number of echoes acquired prior to the acquisition of the echo at the centre of **k**-space following the excitation pulse. For Scheme A, it can be seen that the outer edges of **k**-space are sampled first and the centre of **k**-space is acquired at $T_{\mathrm{E,eff}} = 80\,\hbox {ms}$. For Scheme B, the centre of **k**-space is sampled at $T_{\mathrm{E,eff}}~=~5\,\hbox {ms}$ and the extremities of **k**-space are sampled later in the echo train. Figure 8d shows simulated attenuation data of the signal intensity associated with the water and dodecane according to $T_{2}$ relaxation (Eq. 2) using their respective $T_{2\mathrm{LM}}$ values. Based on this analysis, it is expected that overall the images acquired using Scheme A will have a much greater degree of $T_{2}$ relaxation weighting than the images acquired with Scheme B. Further to this, it is evident that when using Scheme A ($T_{\mathrm{E,eff}}=80~\hbox {ms}$), the signal intensity of the water has a much greater extent of $T_{2}$ weighting associated with it than the dodecane, approximately 22% versus 69% of the signal intensity without relaxation weighting for the water and dodecane, respectively. By contrast, there is much less of a difference in the amount of $T_{2}$ weighting of the water and dodecane signals, using Scheme B ($T_{\mathrm{E,eff}}=5\,\hbox {ms}$), approximately 91 versus 97% of the signal intensity without relaxation weighting for the water and dodecane, respectively.

**Fig. 9:**
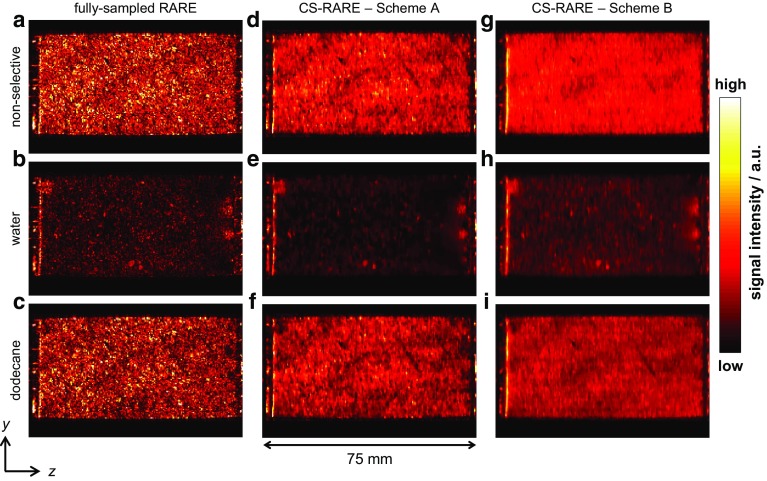
2D slice images in the *zy* plane taken from the 3D images acquired following the injection of 5.8 ml of water into the dodecane-saturated core plug sample. The images shown in the first (**a**–**c**), second (**d**–**f**) and third (**g**–**i**) columns were acquired using the RARE pulse sequence, the CS-RARE pulse sequence using Scheme A and the CS-RARE pulse sequence using Scheme B, respectively. The top row shows the images acquired without chemically-selective preconditioning, whilst the second and third rows show the images acquired with chemically-selective preconditioning to image the water and dodecane, respectively. The voxel resolution is $0.31\,\hbox {mm}\,\times \,0.39\,\mathrm{mm}$ in the *z* and *y* directions, respectively, and the intensity is proportional to the local fluid content

**Fig. 10:**
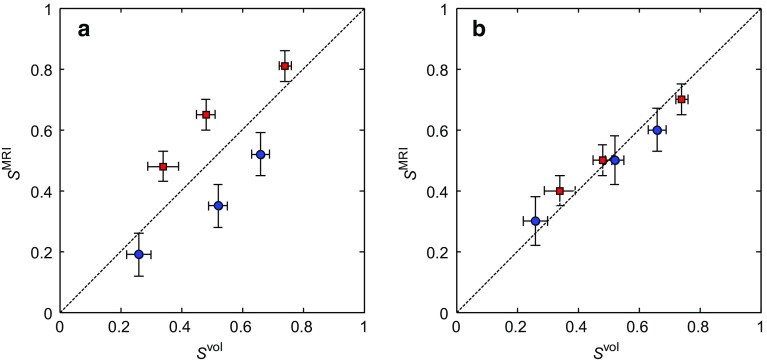
Comparison of the chemically-selective MRI and volumetric determinations of the relative saturations of the dodecane $S_{\mathrm{o}}$ (red squares) and water $S_{\mathrm{w}}$ (blue circles) from the images acquired using the chemically-selective MRI pulse sequence using **a** Scheme A and **b** Scheme B for spatial encoding

Figure 9 shows the 2D slice images in the *zy* plane taken from the series of non-selective and chemically-selective 3D images acquired following the injection of 5.8 ml of water into the dodecane-saturated core plug sample. Figure 9(a–c), (d–f) and (g–i) were acquired using the RARE pulse sequence, the CS-RARE pulse sequence using Scheme A and the CS-RARE pulse sequence using Scheme B, respectively. The top row shows the images acquired without chemically-selective preconditioning, whilst the second and third rows show the images acquired with chemically-selective preconditioning to image the water and dodecane, respectively. In these images, the signal intensity in each voxel is proportional to the local fluid content, which is in turn determined by the local porosity of the rock and relative saturation of the water or dodecane. Due to regularization imposed in the CS reconstruction, the images reconstructed from the under-sampled **k**-space data appear to be smoothed in comparison to the fully sampled counterparts as discussed in Ramskill et al. (2016). Nevertheless, the overall structure of the rock core plug and variation in porosity throughout the sample is still observed. For the CS-RARE acquisitions, it should also be noted that the contrast in the images acquired using Scheme A is somewhat better than the images acquired using Scheme B. This is due to the fact that in Scheme A the extremities of **k**-space (high values of **k**) are sampled earlier in the echo train and the regions of the signal corresponding to the finer details in the image are given more weighting. In Scheme B, low values of **k **are sampled at shorter times in the echo train and therefore lower spatial frequency, coarser-scale features are given more weighting. However, for the in situ monitoring of the multiphase displacement processes that are of interest, the effect of the sampling method on the ability to accurately quantify the relative saturations of the hydrocarbon and aqueous phases is more important than the accuracy of reconstruction of the detailed porosity variations throughout the sample. Using the method described previously (Eqs. 3 and 4), $S_{\mathrm{w}}$ and $S_{\mathrm{o}}$ in the sample have been determined at each stage of the stop-inject-acquire core flood for the CS-RARE measurements employing the two different **k**-space sampling methods (Schemes A and B) and have been compared against the volumetric determinations of $S_{\mathrm{w}}$ and $S_{\mathrm{o}}$ as shown in Fig. 10. It is seen that the values of $S_{\mathrm{w}}$ and $S_{\mathrm{o}}$, as determined from the images acquired using the CS-RARE pulse sequence using Scheme A (Fig. 10a), deviate significantly from the saturation values obtained from volumetric analysis and were found to be accurate to 28% over the range of saturations covered. More specifically, it can be seen that the values of $S_{\mathrm{w}}$ and $S_{\mathrm{o}}$ consistently underestimate and overestimate the expected values, respectively. This is explained by considering the difference in the $T_{2}$ distributions of the water and dodecane (Fig. 7) and the corresponding relaxation time analysis associated with the sampling patterns shown in Fig. 8d. Figure 10b, however, shows that the relative saturation values of the water and dodecane obtained from the CS-RARE pulse sequence using Scheme B, for which the images inherently contain less relaxation weighting, are in much better agreement with the volumetric analysis of the effluent and are accurate to 9% over the range of saturations investigated. This therefore demonstrates the necessity of considering the relaxation behaviour of the different chemical species present in the sample when quantifying the relative saturations in these multiphase systems.

**Fig. 11:**
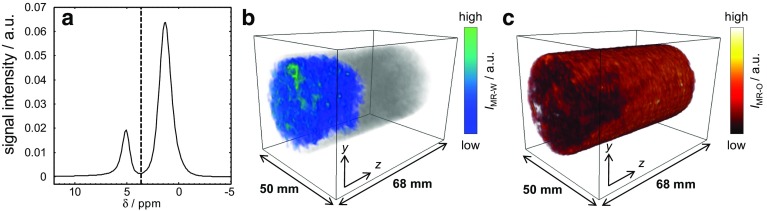
Example NMR and MRI data used to determine the relative water ($S_{\mathrm{w}})$ and oil ($S_{\mathrm{o}})$ saturations in the rock core plug. **a** Bulk NMR spectrum acquired at 0.19 P.V. injected where the signal arising from the water and dodecane is given on the left- and right-hand side of the vertical dotted line, respectively. 3D chemically-selective images of the **b** water and **c** dodecane saturations acquired at 0.20 P.V. injected and 0.22 P.V. injected, respectively

**Fig. 12:**
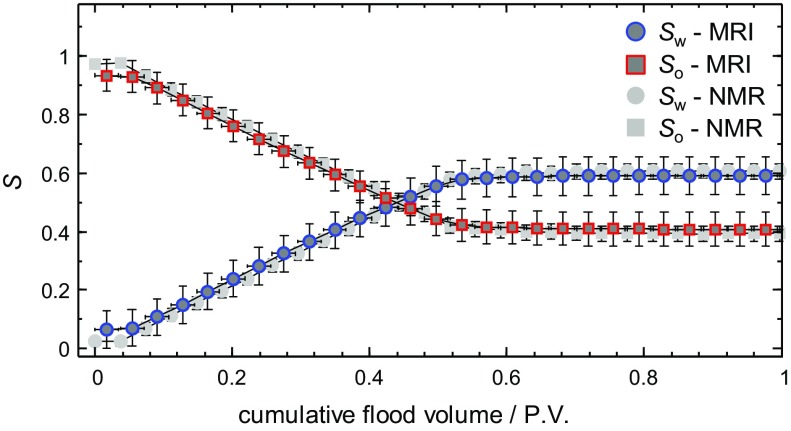
Relative saturations of the water $S_{\mathrm{w}}$ and dodecane $S_{\mathrm{o}}$ in the rock core plug during the core flood experiment as determined from the MRI (foreground) and NMR spectroscopy (background) measurements

For the accurate determination of the relative saturations of oil and water during the dynamic core flood experiment, there is the additional requirement that temporal blurring of the measurement of fluid composition must be minimized whilst still retaining the quantitative nature of the experiment. For the flow rate of $0.025\,\hbox {ml} \ \hbox {min}^{-1}$ used for the core flood experiments, the 2 s recycle delay means that 0.8 ml ($\sim 4\%$ of the pore volume) of water is injected over the course of the 32 min acquisition time. Assuming that the oil–water interface moves through the rock at the interstitial velocity of $1.27\times 10^{-6}\,\hbox {m}\,\hbox {s}^{-1}$ ($0.4\,\hbox {ft}\,\hbox {day}^{-1})$ in the direction of superficial flow, this would equate to movement of $\sim 2$ pixels over the 32 min acquisition time. To assess the effect of $T_{1}$-weighting of the signal intensity incurred by the shorter recycle delay on the quantification of the amount of dodecane and water in the rock, the relative saturations obtained from the images acquired with the shorter and longer recycle delays were determined at the final stage of the inject-stop-acquire core flood. For the images acquired with the shorter recycle delay of 2 s, the dodecane and water saturations were determined to be $S_{\mathrm{o}} = 0.63$ and $S_{\mathrm{w}} = 0.37$, compared to $S_{\mathrm{o}} = 0.60$ and $S_{\mathrm{w}} = 0.40$ as determined from the images acquired with the longer recycle delay of 6 s. Given the estimated errors in the saturation measurements for $S_{\mathrm{o}}$ and $S_{\mathrm{w}}$ it is considered that negligible error has been introduced into the measurement by $T_{1}$-weighting caused by the difference in $T_{1}$ of the water ($T_{1\mathrm{LM}} = 0.7\,\hbox {s}$) and dodecane ($T_{1\mathrm{LM}} = 1.3\,\hbox {s}$). The shorter recycle delay enables a more than three-fold enhancement in the temporal resolution thus allowing for more accurate monitoring of the temporal and spatial dynamics of the displacement process.

### Application to a Dynamic Core Flood

The results from the dynamic core flood experiment of water displacing dodecane from the Estaillades limestone are now presented. To demonstrate the procedure for determining the relative saturations during the core flood, example data acquired around 0.2 P.V. injected in the core flood are shown in Fig. 11. For the NMR spectra (Fig. 11a), the signal intensity associated with the water and dodecane was obtained by integrating the area to the left- and right-hand side of the dashed line ($\delta = 3.5\,\hbox {ppm}$), respectively. For the MRI data, the total signal intensity signal intensities of the water and dodecane were obtained by integrating the signal intensity from the water (Fig. 11b) and dodecane images (Fig. 11c), respectively. Using the integrated signal intensities from the respective measurements, the relative saturations of the water and dodecane were again determined using Eqs. 3 and 4. Figure 12 shows how the relative saturations of the dodecane and water, as determined from the NMR spectroscopy and MRI measurements, change during the core flood experiment. For the NMR spectroscopy measurements, the vertical error bars represent the uncertainty associated with the overlap between the water and dodecane peaks in the spectrum. Horizontal error bars are not visible due to the relatively short acquisition time for these measurements, approximately 1 min. For the MRI data, the vertical error bars represent the maximum uncertainty associated with imperfections in the selective excitations, as determined from a reference measurement prior to the start of the water injection; the horizontal error bars are the uncertainty due to the time required for the acquisition of one set of measurements (i.e. 32 min). It is seen that the relative saturations as determined from the NMR spectra and chemically-selective 3D MRI measurements are in excellent agreement and the residual oil saturations as determined from the two techniques were determined to be $S_{\mathrm{or}}^{\mathrm{NMR}} = 0.39\pm 0.02$ and $S_{\mathrm{or}}^{\mathrm{MRI}}=0.40\pm 0.05$, respectively. Based on these values of $S_{\mathrm{or}}$ and the initial volume of dodecane in the sample, the produced volumes of dodecane were determined to be $13.6\pm 0.4$ and $13.4\pm 1.1$ ml from the NMR spectra and MRI measurements, respectively. From the volumetric analysis of the core flood effluent collected in a measuring cylinder at the outlet of the core holder, the volume of dodecane displaced from the rock at the end of the core flood was $13.2\pm 1.0\,\hbox {ml}$, corresponding to a residual oil saturation of $S_{\mathrm{or}}^{\mathrm{vol}} = 0.41\pm 0.07$, which is in good agreement with both sets of MR measurements. This again confirms the quantitative nature of both the NMR spectroscopy and chemically-selective 3D MRI measurements in determining the relative saturations of the dodecane and water during the core flood.

**Fig. 13:**
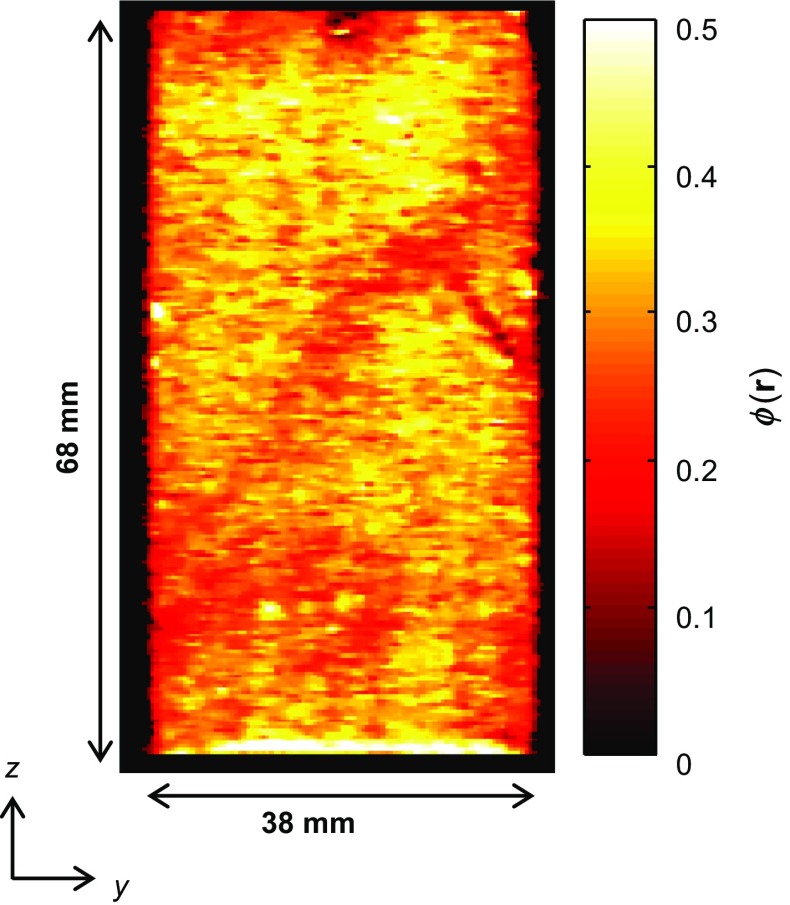
2D slice image of the local porosity ( $\phi$(**r**)) in the *zy* plane taken from the reference 3D image acquired prior to the start of flow when the rock was fully saturated with dodecane. The voxel resolution is $0.31\,\hbox {mm} \,\times \,0.39\,\hbox {mm}$ in the *z* and *y* directions, respectively

**Fig. 14:**
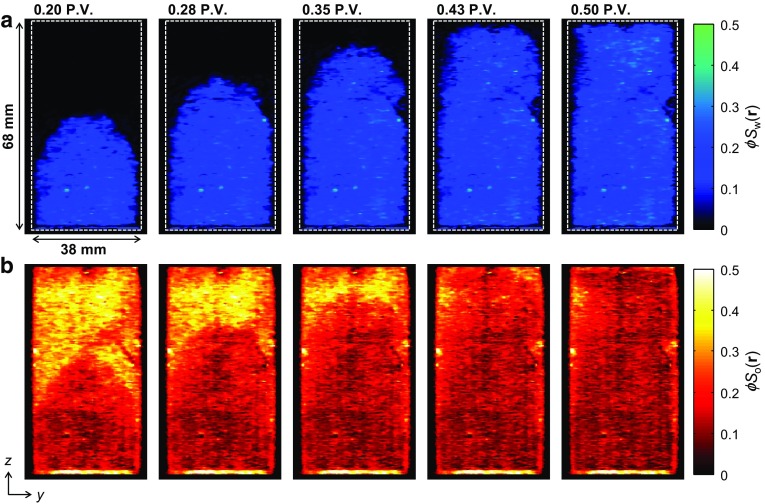
2D slice images in the *zy *plane taken from the 3D images of the **a** water and **b** dodecane distributions acquired independently for different P.V. injected during the core flood experiment. The superficial direction of the injected water flow was in the *z*-direction. The voxel intensity is proportional to the local concentration of the water ($\phi {S}_{\mathrm{w}}$(**r**)) or dodecane ($\phi {S}_{\mathrm{o}}$(**r**))

The bulk saturation measurements are important for determining the residual oil content within the rock, as this can yield information on the overall efficiency of the recovery strategy employed. However, in order to fully characterize the displacement mechanisms in these heterogeneous materials, the temporally and spatially resolved information from the MRI is required. Figure 13 shows a 2D map extracted from the full 3D image acquired at $S_{\mathrm{o}}~=~1$, in which the local signal intensity has been converted to local porosity, $\phi$(**r**), at each spatial position, **r**, using Eqs. 5 and 6:

5 $$\begin{aligned}&\displaystyle \varPhi ( \mathbf{r} )=\frac{I\left( \mathbf{r} \right) }{C}, \end{aligned}$$

6 $$\begin{aligned}&\displaystyle C=\sum I\left( \mathbf{r} \right) \frac{V_{{\mathrm{vox}}} }{V_{\mathrm{T}} }, \end{aligned}$$

where *I*(**r)** is the signal intensity in the voxel at position **r**, *C* is a constant used to convert the local voxel intensities to local porosity, and $V_{\mathrm{vox}}$ and $V_{\mathrm{T}}$ are the volume of an image voxel and total volume of the core plug imaged, respectively. This therefore yields an image in which the intensity is a direct measurement of the local fluid-bearing capacity of the rock. Although at the voxel spatial resolution of the MRI data the individual pores cannot be resolved, the variation in the local porosity throughout the rock is clearly observed. Two-dimensional slice images in the *zy* plane extracted from the full three-dimensional images of the injected water (top panel) and residual dodecane distributions (bottom panel) at different time points during the core flood are shown in Fig. 14a, b, respectively. The intensity scale of the images shown in Fig. 14 is the product of the local porosity (Fig. 13) and relative saturation of either dodecane or water in each voxel, which have been calculated using Eqs. 3 and 4 for each voxel in the image. These images show how the relative quantities of the dodecane and water vary both spatially and temporally within the rock during the displacement.

From the time-series of images shown in Fig. 14, as the water enters the rock, the reduction in the local dodecane content is clearly observed. A stable displacement front of the injected water is seen moving through the core plug along the *z*-axis, i.e. in the direction of superficial flow. Considering the relatively similar viscosities of the water and dodecane used in the present study ($\mu _{\mathrm{w}} = 1.02\,\hbox {cP}$ and $\mu _{\mathrm{o}} = 1.34\,\hbox {cP}$ at $20\,^{\circ }\hbox {C}$), thus giving a mobility ratio ($M={\mu }_{\mathrm{w}}/{\mu }_{\mathrm{o}})$ of $M = 0.76$, a stable displacement front is to be expected in this case. Further, comparing the data shown in Fig. 14 with the bulk saturation measurements shown in Fig. 12, it is seen that the point at which the oil production plateaus and $S_{\mathrm{or}}$ is reached coincides with the water breakthrough, as is shown by the water distribution image at 0.50 P.V. in Fig. 14a. Beyond the water breakthrough point, no further dodecane production occurs as it is now expected that the water will continue to flow through the pore space from which the dodecane has already been displaced, thus bypassing the trapped, residual oil in the rock. This again demonstrates the usefulness of having the spatially resolved information to complement the bulk saturation values. For instance, in an unsteady state relative permeability measurement, as is commonly employed in the SCAL characterization of an asset, the identification of the water breakthrough and amount of oil production beyond this point provide important information with regard to the wetting characteristics of the rock. In the present situation, the fact that oil production appears to stop beyond water breakthrough indicates that this is a water-wetting system (McPhee et al. 2015).

**Fig. 15:**
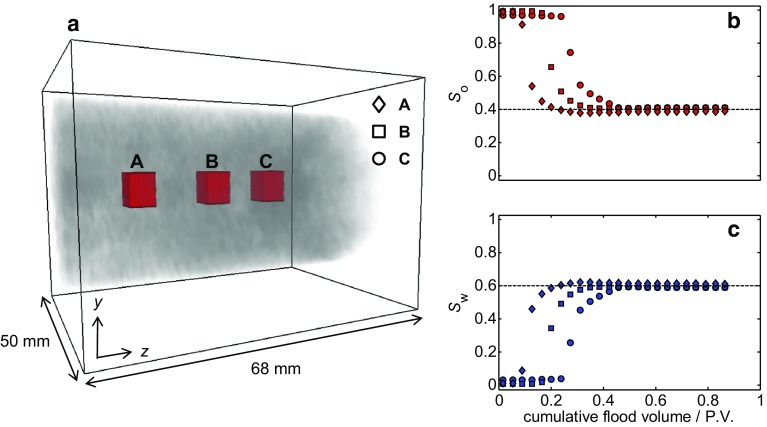
**a** Locations of three approximately $6^{3}\, \hbox {mm}^{3}$ regions of interest (ROI) for which the average **b** $S_{\mathrm{o}}$ and (c) $S_{\mathrm{w}}$ have been determined over the course of the dynamic core flood. The horizontal dashed lines are the overall values of $S_{\mathrm{o}}$ and $S_{\mathrm{w}}$ at the end of the core flood

The local oil and water saturations as a function of the injected volume of water in three approximately $6^{3}\,\hbox {mm}^{3}$ regions of interest (ROI) are shown Fig. 15. This again confirms the uniformity of the displacement of the dodecane across the rock with the residual oil saturations from all three ROI being within 2% of the overall $S_\mathrm{or}$. By having 3D spatially resolved information of the relative saturations in the rock, this provides the capability to only examine the regions of the core plug sample that are representative of displacement in the reservoir and are not influenced by inherent artifacts of the laboratory core flood experiments such as boundary and capillary end effects.

## Conclusions

A novel MRI technique has been demonstrated which has been applied to provide quantitative, 3D spatially resolved and dynamic information of oil and water distributions during an imbibition core flood experiment in a carbonate rock. It has been shown that independent images of the hydrocarbon and aqueous phases can be obtained by exploiting their chemical shift separation in the NMR spectrum. This was achieved by the addition of a chemically-selective preconditioning stage to the RARE image acquisition pulse sequence.

The method was validated by injecting known volumes of water into the initially dodecane-saturated core plug, the relative saturations were then determined using NMR spectroscopy and MRI. From the NMR spectra, excellent agreement with the volumetric analysis has been achieved to an accuracy of 4% over the range of saturation values investigated. For the chemically-selective MRI acquisitions, it was shown that upon optimizing the chemically-selective excitations and by designing the **k**-space sampling pattern to minimize the amount of $T_{2}$ relaxation weighting associated with the image acquisition, agreement between the MRI and volumetric determinations of $S_\mathrm{w}$ and $S_\mathrm{o}$ to within 9% has been achieved over the range of saturations investigated; absolute errors in the MRI saturation measurements being less than 0.04.

The chemically-selective MRI technique was then applied to monitor the continuous injection of water into an initially dodecane-saturated Estaillades core plug at an interstitial velocity of $1.27\times 10^{-6}\,\hbox {m}\,\hbox {s}^{-1}$ ($0.4\,\hbox {ft}\,\hbox {day}^{-1})$. The enhancement in the temporal resolution afforded by using the CS-RARE pulse sequence enabled the dynamics of the displacement front of the injected water to be monitored on a timescale that would not be possible using conventional 3D MRI protocols. The residual oil saturation at the end of the core flood as determined from the MRI measurements was again in excellent agreement with the volumetric determination of the same, $S_{\mathrm{or}}^{\mathrm{MRI}} = 0.40\pm 0.05$ versus $S_{\mathrm{or}}^{\mathrm{vol}} = 0.41\pm 0.07$. The images were also able to provide insights regarding the displacement mechanism occurring within the rock during the dynamic core flood, specifically, that oil production plateaued beyond the water breakthrough point.

The MRI technique demonstrated herein will provide a useful capability for the quantification of structure–transport relationships associated with multiphase displacement processes in complex porous materials encountered in petrophysics research. In future studies, this technique will be used to provide in situ monitoring of the water and oil saturations during capillary pressure and relative permeability measurements for a range of samples with different pore structures and of varying wettabilities.
